# Strengths and Limitations of the Renal Locoregional Perfusion Platform

**DOI:** 10.1016/j.jacbts.2026.101589

**Published:** 2026-06-22

**Authors:** Yosu Luque Rincon

**Affiliations:** a Sorbonne Université, Paris, France; b Hospital Clinic de Barcelona, Barcelona, Spain

I read with great interest the paper by Emmert et al,^1^ who report a fully percutaneous, catheter-based closed loop locoregional perfusion (LRP) concept enabling isolated renal delivery of adeno-associated viral (AAV) vectors in a porcine model. Their work represents a significant contribution to the growing field of organ-targeted advanced therapy delivery and provides one of the most comprehensive demonstrations to date of renal vascular isolation for gene transfer.

A major strength of the study is the rigorous engineering integration of catheter technology, extracorporeal circulation principles, and real-time hemodynamic monitoring. The authors establish a clear “triangle” of flow, pressure, and circuit volume as the key operational determinants of LRP stability and demonstrate their method’s feasibility across both acute and chronic porcine models. The tightness of the LRP compartment, evidenced by up to 69,000-fold higher vector concentration in the isolated circuit compared with systemic blood, highlights the system’s capacity for dramatic vector retention.

Another strength is the authors’ systematic evaluation of AAV serotypes, revealing that AAV5 achieves up to 1,000-fold greater kidney transduction than the other tested capsids. This quantitative comparison, coupled with thorough histological and droplet digital polymerase chain reaction analyses, advances our understanding of capsid-dependent renal tropism. Importantly, the study reports reassuring renal and systemic safety.

Despite its strengths, some limitations warrant consideration. First, the system relies on custom-designed supply and return catheters, a dedicated extracorporeal reservoir, pediatric oxygenator, and vacuum-assisted venous drainage, which may limit immediate clinical implementation in standard interventional radiology. The complexity of circuit management, including frequent adjustments to pump speed, vacuum pressure, vasodilator infusion, and balloon positioning, underscores the need for specialized personnel and environments like cardiac surgery theaters.

*Second, the concept is fundamentally based on non physiological human renal vein pressure,* raising questions about its applicability in patients with impaired renal vascular compliance or chronic kidney disease, who may not tolerate such hemodynamic parameters.

Third, although the authors screened several capsids, these AAVs used green fluorescent protein–encoding genes that have an important tubular autofluorescence that can be confounding.

In conclusion, Emmert et al^1^ provide a compelling demonstration of the power of closed-loop renal perfusion for advanced therapy delivery. Their work establishes essential reference points for circuit stability, capsid selection, and safety. At the same time, the technological complexity and hemodynamic demands of LRP indicate the need for continued exploration of complementary or simplified renal perfusion approaches. The authors’ contribution will undoubtedly serve as a benchmark for future translational innovations in organ-targeted therapeutics.
